# Trauma patient transport to hospital using helicopter emergency medical services or road ambulance in Sweden: a comparison of survival and prehospital time intervals

**DOI:** 10.1186/s13049-023-01168-9

**Published:** 2023-12-16

**Authors:** Oscar Lapidus, Rebecka Rubenson Wahlin, Denise Bäckström

**Affiliations:** 1 Department of Clinical Science, Intervention and Technology, Karolinska Institutet, Stockholm, Sweden; 2 Perioperative Medicine and Intensive Care, Karolinska University Hospital, Huddinge, Sweden; 3 Ambulance Medical Service in Stockholm (AISAB), Stockholm, Sweden; 4 Division of Surgery, Orthopedics and Oncology, Department of Biomedical and Clinical Sciences, Linköping University, Linköping, Sweden; 5 VO Ambulans Och Akut, Region Gävleborg, Sweden

**Keywords:** Prehospital, Trauma, Transport, HEMS, EMS, The Swedish Trauma Registry

## Abstract

**Background:**

The benefits of helicopter emergency medical services (HEMS) transport of adults following major trauma have been examined with mixed results, with some studies reporting a survival benefit compared to regular emergency medical services (EMS). The benefit of HEMS in the context of the Swedish trauma system remains unclear.

**Aim:**

To investigate differences in survival and prehospital time intervals for trauma patients in Sweden transported by HEMS compared to road ambulance EMS.

**Methods:**

A total of 74,032 trauma patients treated during 2012–2022 were identified through the Swedish Trauma Registry (SweTrau). The primary outcome was 30-day mortality and Glasgow Outcome Score at discharge from hospital (to home or rehab); secondary outcomes were the proportion of severely injured patients who triggered a trauma team activation (TTA) on arrival to hospital and the proportion of severely injured patients with GCS ≤ 8 who were subject to prehospital endotracheal intubation.

**Results:**

4529 out of 74,032 patients were transported by HEMS during the study period. HEMS patients had significantly lower mortality compared to patients transported by EMS at 1.9% vs 4.3% (ISS 9–15), 5.4% vs 9.4% (ISS 16–24) and 31% vs 42% (ISS ≥ 25) (p < 0.001). Transport by HEMS was also associated with worse neurological outcome at discharge from hospital, as well as a higher rate of in-hospital TTA for severely injured patients and higher rate of prehospital intubation for severely injured patients with GCS ≤ 8. Prehospital time intervals were significantly longer for HEMS patients compared to EMS across all injury severity groups.

**Conclusion:**

Trauma patients transported to hospital by HEMS had significantly lower mortality compared to those transported by EMS, despite longer prehospital time intervals and greater injury severity. However, this survival benefit may have been at the expense of a higher degree of adverse neurological outcome. Increasing the availability of HEMS to include all regions should be considered as it may be the preferrable option for transport of severely injured trauma patients in Sweden.

## Background

To decrease mortality and morbidity following severe traumatic injuries, several countries utilize helicopter emergency medical services (HEMS) and other rapid response vehicles in addition to regular emergency medical services (EMS) [1, 2]. Investigations regarding the use of HEMS to treat or transport adult patients following major trauma have rendered mixed results, with several authors reporting the existing evidence to be conflicting and of poor quality [2, 3]. Although some investigations have found correlations between HEMS transport and lower mortality, several other studies have found no such benefits [4–7]. While HEMS may be the faster alternative for long-distance transport and in specific circumstances such as during heavy traffic, the external validity of specific distance cur-offs in other trauma systems may be [8]. Previous studies have also shown that while HEMS utilization may be associated with lower mortality, this survival benefit can often not be attributed to faster transport to hospital as HEMS units often have longer prehospital time intervals compared to EMS; instead, these studies suggest any survival benefit is likely due to the higher level of medical expertise of the HEMS crew compared to EMS rather than faster transport to hospital [4–7, 9].

In Sweden, several different HEMS units are in operation in different regions, with the ability of providing advanced medical care in the prehospital setting [10]. Most HEMS units are staffed by physicians, although there are also nurse-staffed HEMS units in some regions [11]. Advanced airway management may be performed may be performed by physicians (typically anesthesiologists) or nurse anesthetists depending on the specific circumstances and the regional guidelines of the EMS system [12]. Several investigations examining prehospital advanced airway management have found that prehospital endotracheal intubation performed by physicians is associated with significantly lower risk of failure compared to non-physician EMS, and that intubation performed by anesthesiologists in the prehospital setting has a high first-pass success rate and low risk of complications [13, 14]. Physicians also allow for more specialized medical interventions in the prehospital setting, which may be beneficial for critically injured patients [15, 16]. Due to conflicting evidence regarding the benefits of prehospital HEMS units, and because the prehospital setting in Sweden may differ significantly from that of the countries in which the use of HEMS has previously been examined, its benefit for the Swedish trauma population remains unclear.

## Aim

To investigate differences in survival and prehospital time for trauma patients in Sweden transported by HEMS compared to road ambulance EMS.

## Ethical considerations

Prior to initiation, this study was approved by the Ethical Review Authority in Sweden (2020-04246, 2022-06727-01).

## Methods

### Setting

Sweden is a northern European country with 10.5 million inhabitants distributed over an area of approximately 529,000 km^2^, with the majority of the population residing in urban clusters [17]. The prehospital trauma system in Sweden consists primarily of nurse-staffed EMS utilizing road ambulance vehicles for patient transport [10, 11]. In urban areas, physician-staffed rapid response vehicles may be dispatched in addition to EMS, which allows for a higher level of expertise and advanced interventions in the prehospital setting [11]. In some regions it is possible to dispatch HEMS units to facilitate faster transport of critically injured patients to hospital, as well as to transport patients where geographical factors may limit the efficacy of transport by road [11, 18]. However, the availability of these resources in subject to regional variation, and as of 2023 HEMS is currently only available in 9 out of 21 regions [10]. There are regional discrepancies in HEMS availability, and dispatch criteria vary between municipalities [10]. In regions where prehospital HEMS units are present, the majority are staffed by physicians, with some notable exceptions such as Region Stockholm which employs a nurse-staffed HEMS service [18]. However, in regions with nurse-staffed HEMS the prehospital system may still allow for physicians to accompany patients during transport to hospital when required [11]. Prehospital physicians staffing HEMS units are required to have completed specialist training in anesthesiology [11].

### The Swedish Trauma Registry

The Swedish Trauma Registry (SweTrau) is a national patient registry encompassing severely injured trauma patients admitted to all reporting hospitals in Sweden. Inclusion criteria are patients admitted to hospital who prompted a trauma team activation, inpatients with a New Injury Severity Score (NISS) > 15 as well as trauma patients transferred to a secondary facility within 7 days of the event. Patients with isolated chronic subdural hematomas are excluded, as well as patients where a trauma team activation is triggered without the presence of a traumatic event. Patient variables are collected according to the Utstein template [19]. In 2022 the registry was reported to have a national coverage of approximately 83% [20]. The Abbreviated Injury Scale (AIS) 2005/2008 was used for injury classification [21].

### Study cohort and outcome

A total of 74,032 trauma patients treated in Sweden during 2012–2022 were identified through SweTrau [20]. Inclusion criteria were age ≥ 15 years and a primary transport method to hospital of either HEMS or road ambulance EMS; exclusion criteria were unknown 30-day mortality or unknown Injury Severity Score (ISS). The primary outcomes were 30-day mortality and Glasgow Outcome Score (GOS) at discharge from hospital (to home or rehab); secondary outcomes were the proportion of severely injured patients (ISS ≥ 16) who triggered a trauma team activation (TTA) on arrival to hospital and the proportion of patients with ISS ≥ 16 and GCS ≤ 8 who were subject to prehospital endotracheal intubation.

Statistical analysis was performed with a significance level of 0.05. The Shapiro–Wilk test was used to determine which variables conformed to normal distribution. Independent samples T-test was used to analyze normally distributed continuous data. Non-parametric tests such as the Mann–Whitney U test and Chi-squared tests were used to analyze non-normally distributed continuous and categorical data respectively. Mean values and standard deviation were determined for normally distributed data; median values and interquartile range were reported for variables with non-normal distribution.

## Results

**Fig. 1:**
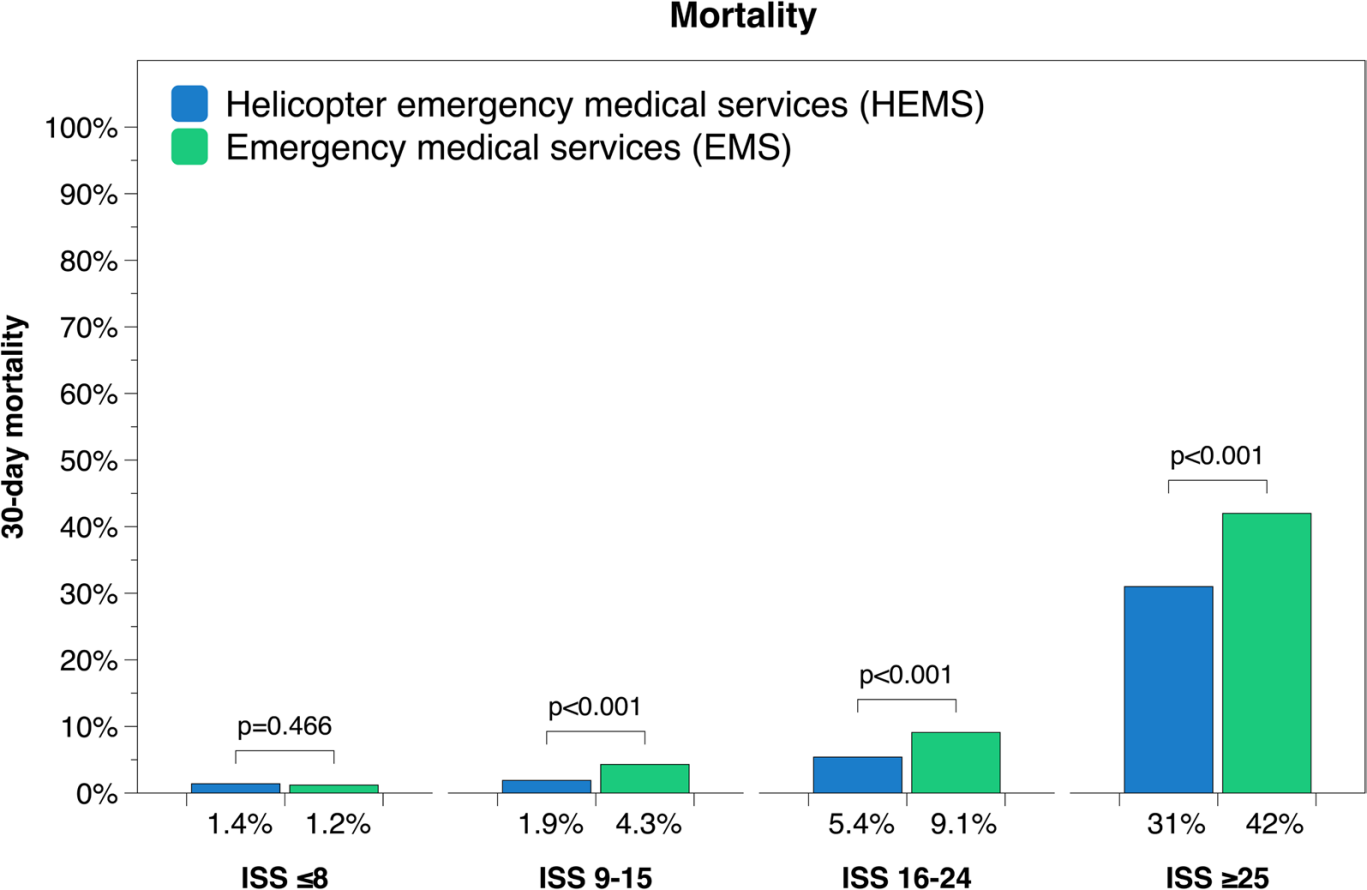
30-day mortality for patients transported by HEMS and EMS

**Fig. 2:**
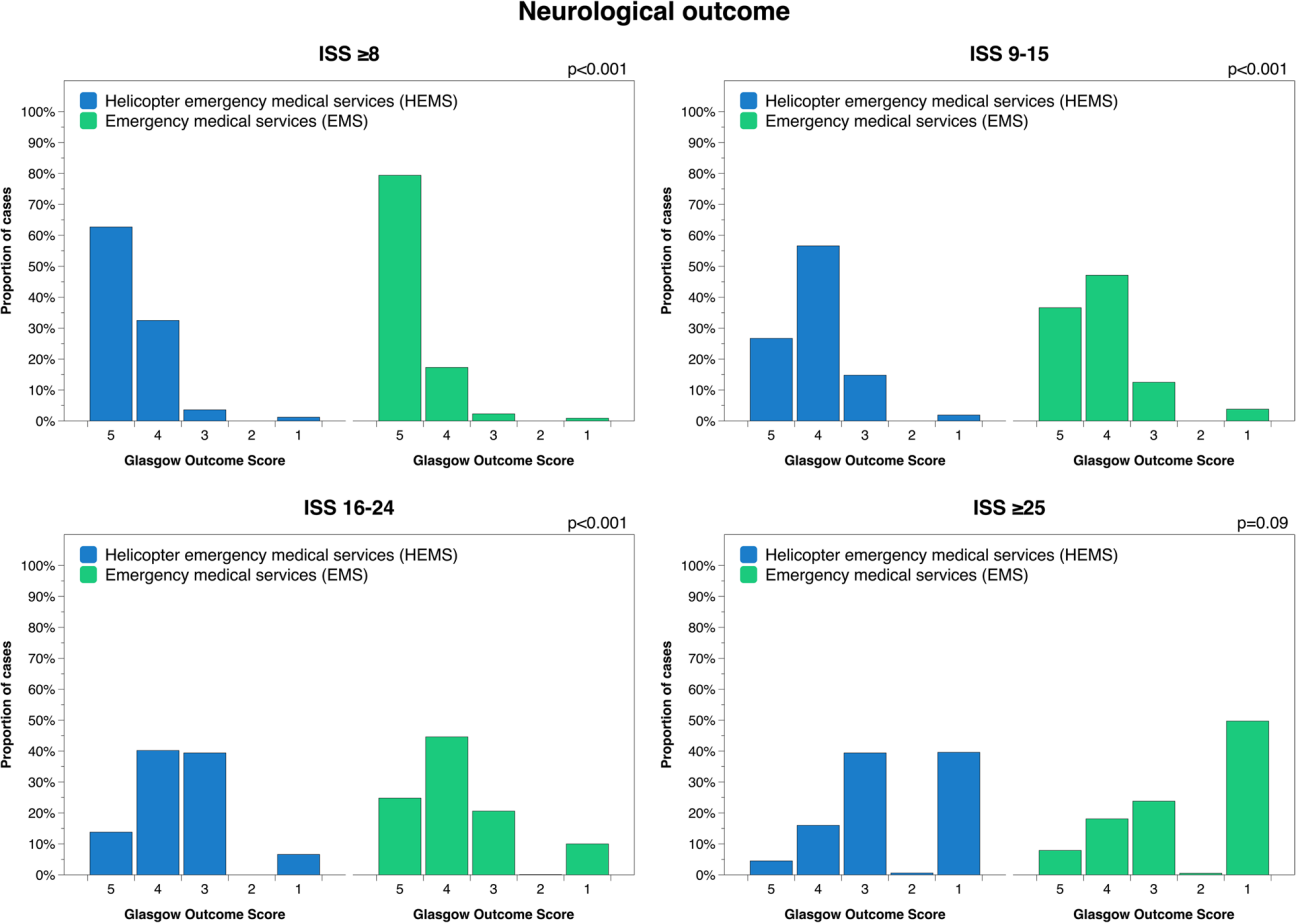
Neurological outcome at discharge to home or rehabilitation for patients transported by HEMS vs EMS

A total of 4529 patients were transported by HEMS during the study period, whereas 69,503 were transported by EMS. Median age was 44 years in both the HEMS and EMS group (Table 1). Overall 30-day mortality was 6.6% for HEMS patients and 4.9% for patients transported by EMS. Adjusted for injury severity, HEMS patients had significantly lower mortality compared to patients transported by EMS at 1.9% vs 4.3% (ISS 9–15), 5.4% vs 9.4% (ISS 16–24) and 31% vs 42% (ISS ≥ 25) (Fig. 1, Table 2); risk ratio was 0.44, 0.60 and 0.75 respectively (p < 0.001). No significant difference was found for patients with ISS ≤ 8 (p = 0.466). However, transport with HEMS was also associated with significantly lower GOS (worse neurological outcome) compared to EMS for all but the most severely injured patients, albeit with similar median values (p < 0.001) (Fig. 2, Table 2).

**Table 1:** Baseline characteristics of the study cohort

Demographics	HEMS *n* = 4529		EMS *n* = 69,503	
Patient characteristics				
Sex (male:female), % (n)	71:29	(3223, 1306)	65:35	(44,988, 24,502)
Age, median (Q1, Q3)	44	(27, 60)	44	(26, 64)
Injury severity	Median	(Q1, Q3)	Median	(Q1, Q3)
ISS ≤ 8	4	(1, 5)	1	(1, 4)
ISS 9–15	10	(9, 13)	10	(9, 13)
ISS 16–24	18	(17, 21)	17	(17, 21)
ISS ≥ 25	30	(27, 41)	29	(26, 35)
Blunt vs penetrating	% Ratio	(n)	% Ratio	(n)
ISS ≤ 8	92:8	(1757, 168)	92:8	(41,981, 3710)
ISS 9–15	92:8	(1116, 91)	91:9	(12,665, 1200)
ISS 16–24	93:7	(668, 50)	92:8	(5076, 447)
ISS ≥ 25	92:8	(615, 56)	90:10	(3817, 402)
Survival				
30-day mortality, % (n)	6.6	(299)	4.9	(3431)
GOS, median (Q1, Q3)	4	(4, 5)	5	(4, 5)
Prehospital time, minutes	Median	(Q1, Q3)	Median	(Q1, Q3)
Total prehospital time	63	(50, 84)	53	(39, 71)
Dispatch to arrival	19	(13, 28)	11	(7, 18)
Time on scene	23	(16, 31)	20	(14, 28)
Scene to hospital	18	(13, 28)	18	(10, 29)

ISS: Injury Severity Score; GOS: Glasgow Outcome Score

**Table 2:** Outcome comparison for patients transported by HEMS vs EMS

Demographics	HEMS		EMS		Significance
30-day mortality	%	(n)	%	(n)	
ISS ≤ 8	1.4	(27)	1.2	(558)	0.466
ISS 9–15	1.9	(23)	4.3	(603)	< 0.001
ISS 16–24	5.4	(39)	9.1	(504)	< 0.001
ISS ≥ 25	31	(210)	42	(1766)	< 0.001
Glasgow outcome score	Median	(Q1, Q3)	Median	(Q1, Q3)	
ISS ≤ 8	5	(4, 5)	5	(5, 5)	< 0.001 ^1^
ISS 9–15	4	(4, 5)	4	(4, 5)	< 0.001 ^2^
ISS 16–24	4	(3, 4)	4	(3, 4)	< 0.001 ^3^
ISS ≥ 25	3	(1, 3)	2	(1, 4)	0.09 ^4^
TTA at hospital	%	(n)	%	(n)	
ISS 16–24	97	(380)	80	(2923)	< 0.001
ISS ≥ 25	99	(369)	85	(2335)	< 0.001
Intubation if GCS ≤ 8	%	(n)	%	(n)	
ISS 16–24	75	(59)	15	(69)	< 0.001
ISS ≥ 25	79	(235)	26	(398)	< 0.001

1. Mean rank HEMS = 18,665, EMS = 22,318; 2. Mean rank HEMS = 5896, EMS = 6417); 3. Mean rank HEMS = 2075, EMS = 2479; 4. Mean rank HEMS = 1988, EMS = 1905

**Fig. 3:**
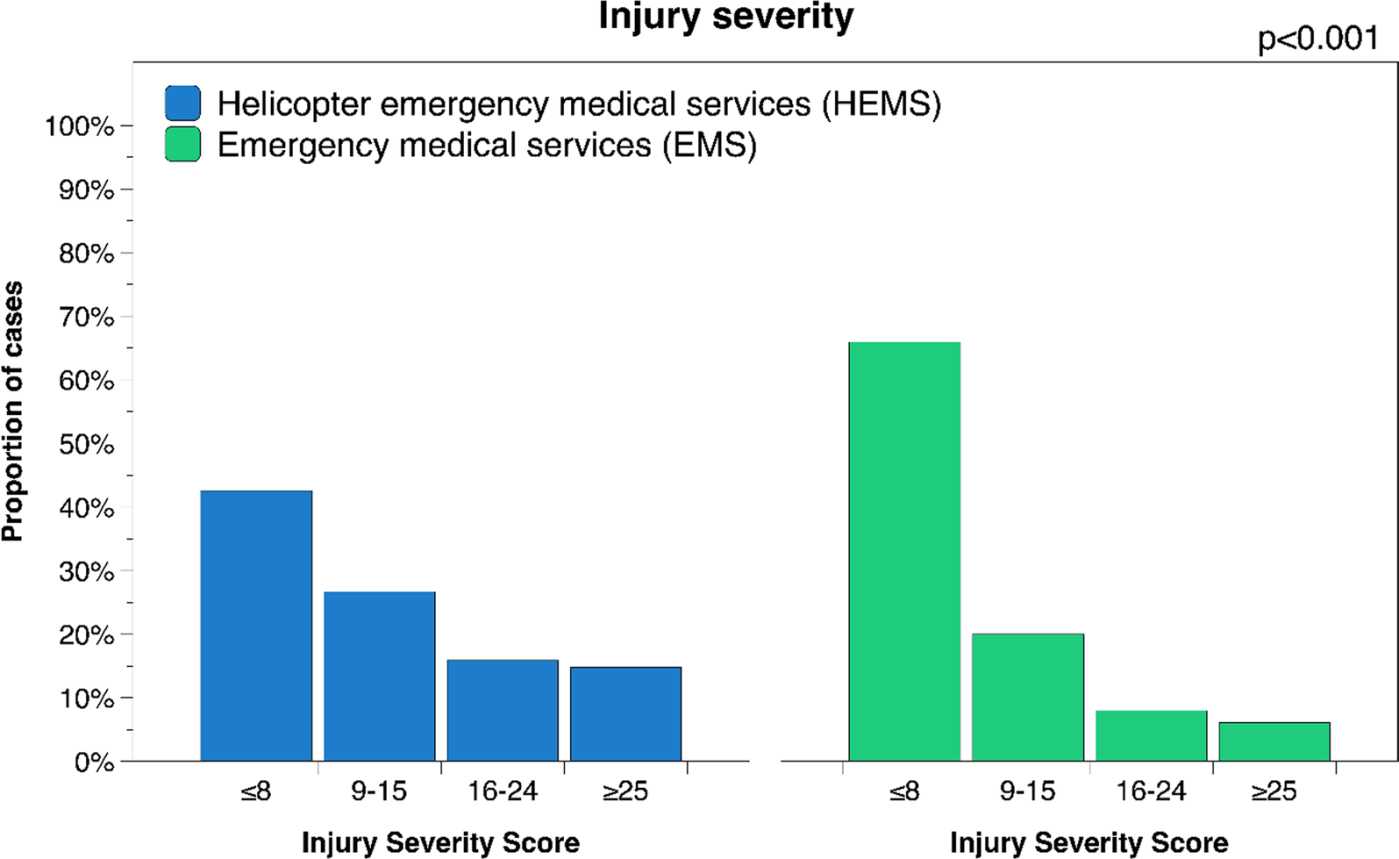
Injury severity distribution for patients transported by HEMS vs EMS

The rate of in-hospital TTA for severely injured patients was significantly higher for patients transported by HEMS at 97% vs 80% (ISS 16–24) and 99% vs 85% (ISS ≥ 25) compared to patients transported by EMS (p < 0.001) (Table 2). The proportion of patients with ISS ≥ 16 and GCS ≤ 8 who were subject to endotracheal intubation in the prehospital setting was also significantly higher for patients transported by HEMS at 75% vs 15% (ISS 16–24) and 79% vs 26% (ISS ≥ 25) compared to patients transported by EMS (p < 0.001) (Table 2). There was a significant trend towards higher ISS in the HEMS group compared EMS in the cohort overall, as well as within each separate injury severity group (p < 0.001) (Fig. 3, Table 1).

Total prehospital time, as well as the duration of all prehospital time intervals, was longer for patients transported by HEMS compared to EMS in all injury severity groups (Table 3). Total prehospital time was averaged 63 min for HEMS patients and 53 min for patients transported by EMS (p < 0.001); similar differences were also found for time from dispatch to arrival on scene (19 vs 11 min) and time on scene (23 vs 20 min) (Table 1, Table 3). The median time from scene to hospital was 18 min in both groups, but statistical analysis of the distributions showed HEMS transport was associated with longer time from scene to hospital (Table 3).

**Table 3:** Prehospital time for patients transported by HEMS vs EMS

Demographics	HEMS		EMS		Significance
ISS ≤ 8					
Total prehospital time	62	(48, 83)	53	(40, 71)	< 0.001
Dispatch to scene	19	(13, 29)	12	(7, 18)	< 0.001
Time on scene	21	(15, 30)	20	(14, 27)	< 0.001
Scene to hospital^1^	18	(12, 27)	18	(10, 29)	0.002 ^1^
ISS 9–15					
Total prehospital time	64	(52, 85)	55	(40, 73)	< 0.001
Dispatch to scene	20	(14, 29)	11	(7, 18)	< 0.001
Time on scene	23	(16, 32)	21	(14, 29)	< 0.001
Scene to hospital^2^	18	(13, 28)	18	(11, 29)	0.006 ^2^
ISS 16–24					
Total prehospital time	66	(51, 86)	52	(39, 70)	< 0.001
Dispatch to scene	20	(13, 29)	11	(7, 11)	< 0.001
Time on scene	23	(17, 33)	20	(14, 20)	< 0.001
Scene to hospital	19	(13, 29)	17	(10, 28)	< 0.001
ISS ≥ 25					
Total prehospital time	61	(47, 85)	47	(34, 63)	< 0.001
Dispatch to scene	17	(12, 26)	10	(6, 15)	< 0.001
Time on scene	24	(17, 33)	19	(12, 25)	< 0.001
Scene to hospital	17	(11, 28)	15	(9, 25)	< 0.001

1. Mean rank HEMS = 24,670, EMS = 23,697; 2. Mean rank HEMS = 7854, EMS = 7495

## Discussion

The present study aimed to investigate differences in survival and prehospital time for trauma patients in Sweden transported by HEMS compared to EMS and found that moderately-severely injured HEMS patients have significantly lower injury-adjusted mortality compared to patients transported by EMS. However, as HEMS patients also had lower GOS at discharge from hospital, it may be that the higher survival rate was at the expense of worse neurological outcome. These results are consistent with several previous studies reporting significant associations between HEMS transport and lower mortality for patients with severe trauma [4, 22–24]. Similarly, a study by Biewener et al. found that HEMS transport was associated with a significantly lower risk of death for patients with severe blunt trauma in Germany [23]. The findings of the present study are in concordance with a study by Nasser et al. which found that HEMS transport of patients was associated with a 32% decrease in the odds-ratio of mortality following penetrating trauma; however, although the present study examined patients with predominantly blunt injuries, this may suggest the presence of a time-critical component in both cohorts [22].

One potential explanation for the lower mortality in the HEMS group is the increased level of expertise provided by the HEMS crew, as well as the ability to provide more advanced treatments and prioritize meaningful interventions in the early phase of trauma care. Although not specifically examined in the present study, HEMS patients were likely more frequently exposed to a high level of medical expertise in the prehospital setting due to the inherent nature and staffing policies of the various HEMS services in Sweden, which may be beneficial as physicians bring a higher level of expertise regarding medical interventions such as advanced airway management [24]. This is evident from the proportion of patients with ISS ≥ 16 and GCS ≤ 8 who were treated with endotracheal intubation in the prehospital setting, which was significantly higher for HEMS patients compared to patients transported by EMS. It is not known whether this discrepancy is due to a higher level of precaution in the HEMS group generated by the inherent difficulties of helicopter transport or because of a difference in skill availability. As the majority of HEMS units in Sweden are staffed by physicians who have completed specialist training in anesthesiology who have also been shown to have higher first-pass success rate compared to nurse anesthetists, it may be that HEMS crew feel more comfortable providing advanced airway management to severely injured patients in the prehospital setting [12].

Previous studies have shown that the use of prehospital physicians may be beneficial for critically ill patients, supporting the notion that physician expertise in the prehospital setting may contribute to improved outcomes for trauma patients [15, 16]. Past authors have reported mixed assessments of whether any survival benefit is related to decreased transport time [8, 25–27]. In the present study, HEMS patients had significantly shorter prehospital time intervals compared to patients transported by EMS across all categories of injury severity. While this may be the result of an selection bias towards patients with longer transport distances in the HEMS group, it is also possible that this time discrepancy is inherent to the use of a helicopter, such as additional time consumed because of delayed dispatch compared to EMS, or longer time spent loading/unloading the patient during departure and arrival. While no investigations have directly compared the transport time of HEMS and EMS in Sweden, the prehospital time intervals of the present study were generally shorter than what has previously been reported from the HEMS unit operated by the Swedish Air Ambulance, which found a median mission time of 90 min (compared to 63 min in the present study); this discrepancy is likely due to the different geographic contexts in which the studies were performed [28]. However, in the present study total prehospital time and all constituent time intervals were still longer for patients transported by HEMS compared to EMS. This may suggest the survival benefit is not the result of a time-saving advantage, which is in concordance with several previous studies reporting patients transported by HEMS have lower mortality despite longer prehospital time intervals [8, 9, 29]. Brown et al. found this survival benefit to be concentrated to transport times between 6 and 30 min, which appropriately reflects the observed transport time of the present study [9]. Although numerous studies have stressed the general importance of timely transport to hospital following major trauma, multiple investigations of prehospital critical care teams with a higher degree of medical expertise have failed to show any association between prehospital time and mortality, with some reporting longer prehospital time to be associated with decreased mortality [30–34]. Although these results are observational, this may reflect a clinical reality where rapid access to advanced medical care is the important factor, whether it be via transporting the patient to hospital or by bringing the advanced care to the patient in the prehospital setting [35].

It is possible that the lower mortality of patients transported by HEMS is due to a selection bias this study failed to adjust for, as the most critically injured patients may have been promptly sent to hospital with the first available method of transport (often EMS because of shorter response time), contributing to higher mortality in the EMS group. Another possibility is that patients with obvious critical injuries are identified as seriously injured in the prehospital setting, consequently prompting dispatch of HEMS, whereas patients with occult injuries of equal severity may not be as readily identified to be seriously injured and are instead transported by EMS. The same reasoning may explain the higher proportion of TTA for patients transported by HEMS as this alone may be a sign of severe injury. Regardless, lower mortality despite higher injury severity and longer prehospital transport time combined with lower rates of under-triage on arrival to hospital suggests HEMS transport may be the preferrable option for transport of severely injured trauma patients in Sweden.

### Limitations

While the results of this study may be of interest for the Swedish trauma population, there are a few limitations which should be acknowledged. This study was performed in the context of the Swedish prehospital trauma system and examined cases from all SweTrau-reporting hospitals. While this study may reflect the potential benefits of HEMS transport for trauma patients in Sweden, the external validity of these results remains uncertain. As previously showed in a meta-analysis by Galvagno et al., several studies have also failed to demonstrate any survival benefit associated with HEMS compared to EMS, likely due to these studies being performed in a diversity of prehospital trauma systems [2].

As HEMS patients were generally more seriously injured than patients transported by HEMS, the finding of lower mortality in this group is encouraging. However, these patients also had lower GOS overall (worse neurological outcome), and those who survived generally did so with a higher degree of neurological disability. This finding may be directly associated with the higher rate of survival for the most severely injured patients, where some degree neurological deficit may be inevitable, but could also be due to the inherent nature of some types of injuries which are more prone to prompt HEMS dispatch such as traumatic brain injuries. One limitation of the present study is that no attempts were made to adjust for injury type or baseline neurological function; in addition, while GOS is measure and reported to SweTrau in accordance with the Utstein template, it may still be subject to variation in individual assessment [19].

As the present study did not exclude patients who were dead on arrival to hospital, this may have affected the mortality of both groups. These patients may have been preferentially transported by EMS, contributing to higher mortality in this group, but likely constitute only a small fraction of examined cases. In addition, the present study included cases from all hospitals reporting to SweTrau whether or not there was access to a prehospital HEMS unit, which introduces a selection bias as some patients are obliged to be transported by EMS. The significance of this bias is debatable when making injury-adjusted comparisons. However, this was done based on ISS groups, hence this is merely a crude form of injury-adjusted mortality and may warrant further investigations utilizing more sophisticated methodology. Whether or not patients transported by EMS had a theoretical accessibility to HEMS may also influence this interpretation, as patients being treated in a certain region which utilizes HEMS is not a definitive measure of whether HEMS resources were available for that patient. Future investigations only examining regions where HEMS resources are present may be of interest and should ideally examine a propensity-matched trauma cohort. Weather and other external circumstances may significantly affect the availability of HEMS, as well as the number of severely injured patients at any one point in time. Because this study included multiple independently operated HEMS units in Sweden, the availability of ground EMS units due to geographic factors may also vary between trauma systems; likewise, this study did not differentiate between patients treated by physician-staffed and nurse-staffed HEMS units, hence further investigations further isolated investigations in different trauma systems may be of value for detailed evaluation of the performance of HEMS vs EMS in Sweden.

## Conclusion

Trauma patients transported to hospital by HEMS had significantly lower mortality compared to those transported by EMS, despite longer prehospital time intervals and greater injury severity. However, this survival benefit may have been at the expense of a higher degree of adverse neurological outcome. Increasing the availability of HEMS to include all regions should be considered as it may be the preferrable option for transport of severely injured trauma patients in Sweden.

## Data Availability

The data will not be published but is available from the corresponding author at reasonable request.

## Abbreviations

HEMS: Helicopter emergency medical services
EMS: Emergency medical services
SweTrau: The Swedish Trauma Registry
AIS: Abbreviated injury scale
ISS: Injury Severit Score
NISS: New injury severity score
GOS: Glasgow outcome score
TTA: Trauma team activation
GCS: Glasgow coma score

## References

[1] Rugg C, Woyke S, Ausserer J, Voelckel W, Paal P, Strohle M (2021). Analgesia in pediatric trauma patients in physician-staffed Austrian helicopter rescue: a 12-year registry analysis. Scand J Trauma Resusc Emerg Med.

[2] Galvagno SM, Jr., Sikorski R, Hirshon JM, Floccare D, Stephens C, Beecher D, et al. Helicopter emergency medical services for adults with major trauma. Cochrane Database Syst Rev. 2015(12):CD009228. 10.1002/14651858.CD009228.pub3 PMC8627175 26671262

[3] Moore L, Champion H, Tardif PA, Kuimi BL, O'Reilly G, Leppaniemi A (2018). Impact of trauma system structure on injury outcomes: A systematic review and meta-analysis. World J Surg.

[4] Andruszkow H, Lefering R, Frink M, Mommsen P, Zeckey C, Rahe K (2013). Survival benefit of helicopter emergency medical services compared to ground emergency medical services in traumatized patients. Crit Care.

[5] Nabeta M, Murotani K, Kannae M, Tashiro K, Hirayu N, Morita T (2021). Comparison of physician-staffed helicopter with ground-based emergency medical services for trauma patients. Am J Emerg Med.

[6] Beaumont O, Lecky F, Bouamra O, Surendra Kumar D, Coats T, Lockey D (2020). Helicopter and ground emergency medical services transportation to hospital after major trauma in England: a comparative cohort study. Trauma Surg Acute Care Open.

[7] Blasius FM, Horst K, Brokmann JC, Lefering R, Andruszkow H, Hildebrand F (2021). Helicopter emergency medical service and hospital treatment levels affect survival in pediatric trauma patients. J Clin Med.

[8] Chen X, Gestring ML, Rosengart MR, Peitzman AB, Billiar TR, Sperry JL (2018). Logistics of air medical transport: When and where does helicopter transport reduce prehospital time for trauma?. J Trauma Acute Care Surg.

[9] Brown JB, Gestring ML, Guyette FX, Rosengart MR, Stassen NA, Forsythe RM (2016). Helicopter transport improves survival following injury in the absence of a time-saving advantage. Surgery.

[10] Sveriges prehospitala akutsjukvård - nulägesbild, bedömning och utvecklingsförslag. Socialstyrelsen; 2023. Contract No.: 2023–2–8337.

[11] Lennart Christiansson JL, Eriksson B, Fläring U, Sundin P, Sandström E. Prehospital traumasjukvård – Delprojekt till Socialstyrelsens regeringsuppdrag. att utarbeta ett planeringsunderlag för traumavård. Socialstyrelsen; 2015.

[12] Gellerfors M, Fevang E, Backman A, Kruger A, Mikkelsen S, Nurmi J (2018). Pre-hospital advanced airway management by anaesthetist and nurse anaesthetist critical care teams: a prospective observational study of 2028 pre-hospital tracheal intubations. Br J Anaesth.

[13] Rognas L, Hansen TM, Kirkegaard H, Tonnesen E (2013). Pre-hospital advanced airway management by experienced anaesthesiologists: a prospective descriptive study. Scand J Trauma Resusc Emerg Med.

[14] Lossius HM, Roislien J, Lockey DJ (2012). Patient safety in pre-hospital emergency tracheal intubation: a comprehensive meta-analysis of the intubation success rates of EMS providers. Crit Care.

[15] Rognas L, Hansen TM, Kirkegaard H, Tonnesen E (2013). Refraining from pre-hospital advanced airway management: a prospective observational study of critical decision making in an anaesthesiologist-staffed pre-hospital critical care service. Scand J Trauma Resusc Emerg Med.

[16] Rognas L, Hansen TM, Kirkegaard H, Tonnesen E (2014). Anaesthesiologist-provided prehospital airway management in patients with traumatic brain injury: an observational study. Eur J Emerg Med.

[17] Statistics Sweden – Statistical Database. Accessed 2022–12–23.

[18] Janne Kautto AE, Söderberg P. Direktiv 10:4—Utlarmning av ambulanshelikopter. Web; 2023 2023–05–16. Contract No.: HSN 2023–0496

[19] Ringdal KG, Coats TJ, Lefering R, Di Bartolomeo S, Steen PA, Roise O (2008). The Utstein template for uniform reporting of data following major trauma: a joint revision by SCANTEM, TARN, DGU-TR and RITG. Scand J Trauma Resusc Emerg Med.

[20] The Swedish Trauma Registry (SweTrau). Registercentrum Syd

[21] Gennarelli T, Wodzin E. Association for the Advancement of Automotive Medicine (2008) Abbreviated injury scale 2005: update 2008. Association for the Advancement of Automative Medicine, Barrington. 2008.

[22] Nasser AAH, Khouli Y (2020). The impact of prehospital transport mode on mortality of penetrating trauma patients. Air Med J.

[23] Biewener A, Aschenbrenner U, Rammelt S, Grass R, Zwipp H (2004). Impact of helicopter transport and hospital level on mortality of polytrauma patients. J Trauma.

[24] Garner A, Rashford S, Lee A, Bartolacci R (1999). Addition of physicians to paramedic helicopter services decreases blunt trauma mortality. Aust N Z J Surg.

[25] Schneider AM, Ewing JA, Cull JD (2021). Helicopter transport of trauma patients improves survival irrespective of transport time. Am Surg.

[26] Sborov KD, Gallagher KC, Medvecz AJ, Brywczynski J, Gunter OL, Guillamondegui OD (2020). Impact of a new helicopter base on transport time and survival in a rural adult trauma population. J Surg Res.

[27] Meyer MT, Gourlay DM, Weitze KC, Ship MD, Drayna PC, Werner C (2016). Helicopter interfacility transport of pediatric trauma patients: Are we overusing a costly resource?. J Trauma Acute Care Surg.

[28] Kornhall D, Naslund R, Klingberg C, Schiborr R, Gellerfors M (2018). The mission characteristics of a newly implemented rural helicopter emergency medical service. BMC Emerg Med.

[29] Stewart KE, Cowan LD, Thompson DM, Sacra JC, Albrecht R (2011). Association of direct helicopter versus ground transport and in-hospital mortality in trauma patients: a propensity score analysis. Acad Emerg Med.

[30] Bjorkman J, Setala P, Pulkkinen I, Raatiniemi L, Nurmi J (2022). Effect of time intervals in critical care provided by helicopter emergency medical services on 30-day survival after trauma. Injury.

[31] Maddock A, Corfield AR, Donald MJ, Lyon RM, Sinclair N, Fitzpatrick D (2020). Prehospital critical care is associated with increased survival in adult trauma patients in Scotland. Emerg Med J.

[32] Melendez-Lugo JJ, Caicedo Y, Guzman-Rodriguez M, Serna JJ, Ordonez J, Angamarca E (2020). Prehospital damage control: the management of volume, temperature and bleeding!. Colomb Med (Cali)..

[33] Butler DP, Anwar I, Willett K (2010). Is it the H or the EMS in HEMS that has an impact on trauma patient mortality? A systematic review of the evidence. Emerg Med J.

[34] Pham H, Puckett Y, Dissanaike S (2017). Faster on-scene times associated with decreased mortality in Helicopter Emergency Medical Services (HEMS) transported trauma patients. Trauma Surg Acute Care Open.

[35] Chen X, Gestring ML, Rosengart MR, Billiar TR, Peitzman AB, Sperry JL (2018). Speed is not everything: Identifying patients who may benefit from helicopter transport despite faster ground transport. J Trauma Acute Care Surg.

