# Correction: Trauma patient transport to hospital using helicopter emergency medical services or road ambulance in Sweden: a comparison of survival and prehospital time intervals

**DOI:** 10.1186/s13049-024-01173-6

**Published:** 2024-02-19

**Authors:** Oscar Lapidus, Rebecka Rubenson Wahlin, Denise Bäckström

**Affiliations:** 1 Department of Clinical Science, Intervention and Technology, Karolinska Institutet, Stockholm, Sweden; 2 Perioperative Medicine and Intensive Care, Karolinska University Hospital, Huddinge, Sweden; 3 Ambulance Medical Service in Stockholm (AISAB), Stockholm, Sweden; 4 Division of Surgery, Orthopedics and Oncology, Department of Biomedical and Clinical Sciences, Linköping University, Linköping, Sweden; 5 VO Ambulans Och Akut, Region Gävleborg, Sweden

**Correction: Scand J Trauma Resusc Emerg Med (2023) 31:101** 10.1186/s13049-023-01168-9

After publication of this article [1], the authors reported that in Fig. 3 the text along the y-axis is incorrect; the figure should have appeared as shown below.

**Fig. 3:**
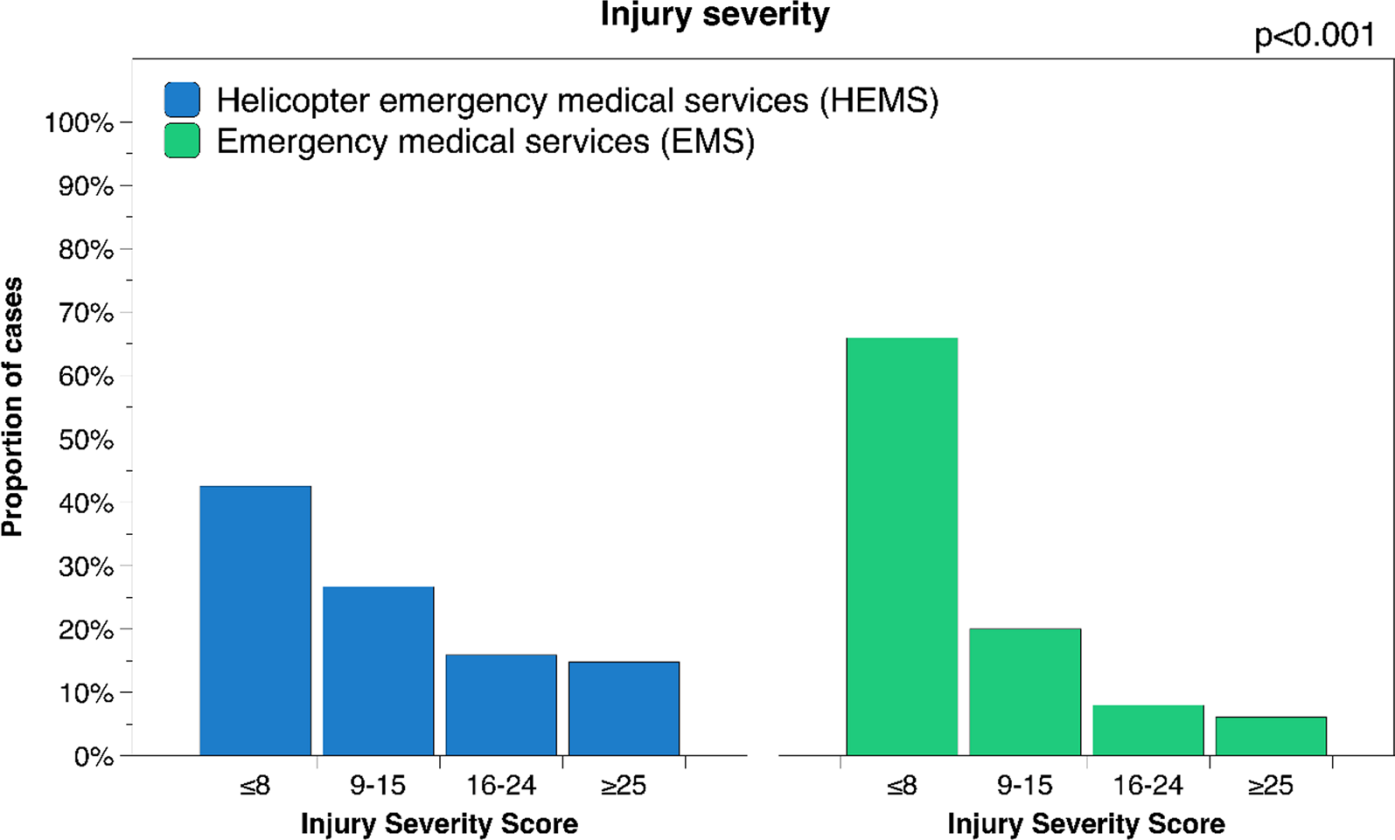
Injury severity distribution for patients transported by HEMS vs EMS

The original article [1] has been corrected.
